# Membrane Protein Insertion in Cells: Principles, Pathways, and Quality Control

**DOI:** 10.1021/acs.chemrev.5c01033

**Published:** 2026-05-28

**Authors:** Hadas Peled-Zehavi, Reut Yemini, Nir Fluman

**Affiliations:** Department of Biomolecular Sciences, 34976 Weizmann Institute of Science, 234 Herzl St. PO Box 26, Rehovot 7610001, Israel

## Abstract

Integral membrane proteins comprise at least a quarter of every proteome. To fold and function properly, these proteins must insert into the correct membrane with the proper topology and orientation. Although their transmembrane helices are chemically compatible with the bilayer, insertion inside cells is not a simple spontaneous event. It must occur in a crowded environment, at the correct membrane, and it often involves challenges such as transferring hydrophilic segments across the bilayer or accommodating helices that are only marginally compatible with it. Cells therefore rely on dedicated systems that direct membrane proteins to the membrane, mediate their insertion, and monitor the process to ensure that it occurs correctly. This review outlines the principles that govern membrane protein insertion in cells and explains how transmembrane sequence features interact with the machineries that mediate their entry into the bilayer. We highlight the major insertion systems of bacteria and eukaryotes and the auxiliary factors that support them, and describe how these pathways accommodate the broad range of membrane protein architectures found in cells, from single-pass proteins to complex multispanning transporters. We also discuss how cells maintain accuracy when insertion fails, through mechanisms that detect and resolve misinsertion. Together, these concepts present membrane protein insertion as a coordinated, adaptable, and safeguarded process, shaped by the interplay between sequence properties, membrane environments, and the machinery responsible for building the membrane proteome.

## Graphical abstract

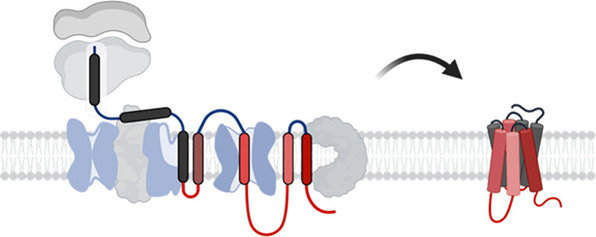

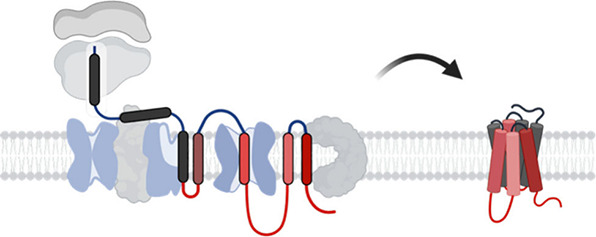

## 1 Introduction

In nearly every living cell, thousands to tens of thousands of membrane proteins (MPs) are produced every minute.^1^ This mass production supports the many vital roles that MPs play in signal transduction, metabolism, nutrient and ion transport, catalysis, and other essential cellular processes. However, making even a single functional MP is a daunting task. To become functional, MPs must reach the correct membrane, insert into it, fold into a precise three-dimensional structure, and assemble with the appropriate protein partners. While each of these steps is thermodynamically favorable and could, in principle, occur spontaneously,^2–5^ in cells these processes are error-prone and therefore tightly mediated and monitored by intricate molecular machineries.^4–6^

This review focuses on one essential step that all integral MPs undergo: insertion of the protein into the membrane. We concentrate on α-helical MPs, which constitute the vast majority of MPs.^7^ Beta-barrel MPs, which are inserted by distinct mechanisms, are covered in another review in this issue.^8^

Membrane insertion is crucial not only for anchoring MPs at their correct cellular location, but also for residing in the correct physicochemical environment required for MP folding (for recent reviews, see^2,3,9^ ). For example, insertion into the membrane induces the α-helical structure of transmembrane helices (TMs) and orients them roughly perpendicular to the membrane plane. These events form the starting point for the three-dimensional packing of the TMs into a functional fold, a process also shaped by the lipid bilayer’s properties.^9^ Thus, insertion is a foundational first step in MP folding.

From a purely chemical perspective, insertion may appear simple, as hydrophobic TMs are favored within membranes. In cells, however, it is an intricate, multifactorial process that affects both genetic disease mechanisms and MP evolution. In this review, we aim to link the chemistry, biochemistry, and cell biology of membrane protein insertion, and explain the logic behind the cellular systems that mediate and surveil this essential step.

## 2 The Chemistry of Membrane Insertion: Chemical Rules, Cellular Realities

Whether a particular polypeptide segment inserts into the membrane as a transmembrane helix depends primarily on thermodynamics. Specifically, how energetically favorable it is for the segment to reside in the membrane, compared to the aqueous cytosol or the intermediate environment of the membrane interface^3,10–12^ (Figure 1a). The driving force for insertion stems from chemical complementarity between the TM segment and the hydrophobic core of the membrane.^3,10,13,14^ In the simplest terms, TMs are hydrophobic stretches of ∼20 amino acids which, when folded into an α helix, are just long enough to span the ∼30 Å hydrophobic core of a lipid bilayer.^15^ Within the membrane, polar groups, whether from the backbone or side chains, are energetically disfavored, as the membrane cannot provide hydrophilic partners for hydrogen bonding or ionic interactions. The α-helical conformation resolves this problem by internally satisfying the backbone hydrogen-bonding potential, while exposing hydrophobic side chains to interact favorably with the surrounding lipids.^3^

**Figure 1:**
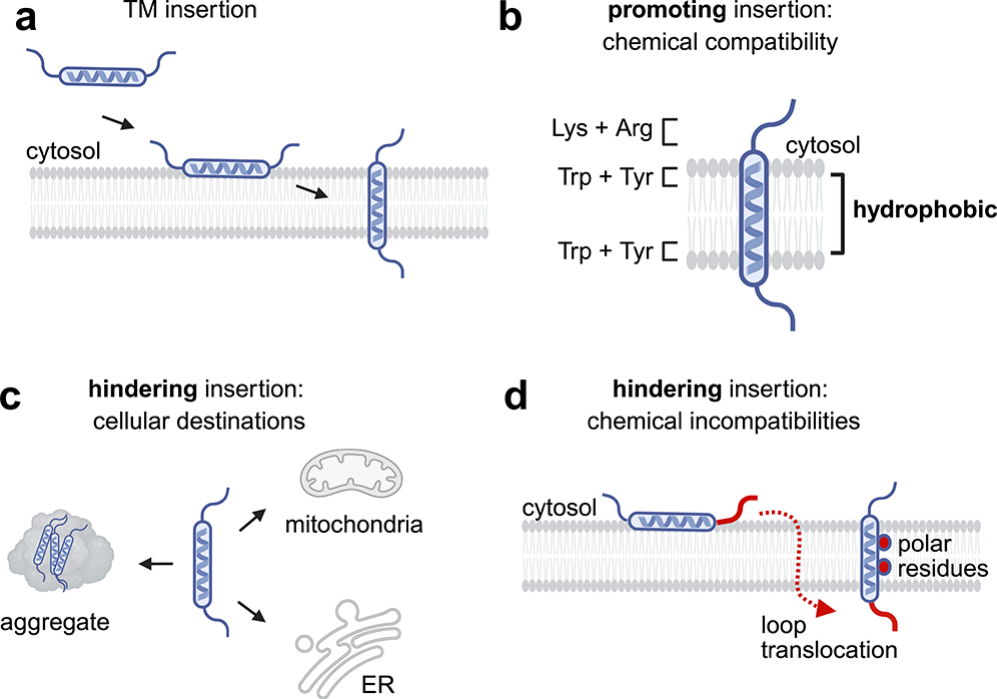
**Determinants and barriers of TM insertion.** a. Steps of TM insertion. TMs and their flanking sequences are synthesized in the aqueous cytosol. They first arrive at the membrane, may transiently adsorb to its surface, and then insert into the bilayer. b. Sequence determinants that promote insertion. Insertion is driven by compatibility between the TM and the membrane. Hydrophobicity provides the dominant contribution; additional stabilization arises from aromatic residues (Trp, Tyr) at the lipid interface and from positively charged residues (Lys, Arg) on the cytosolic flanking sequence. c. Multiple potential cellular destinations hinder TM insertion by competing with delivery to the correct membrane. Newly synthesized TMs must be targeted to the correct membrane while avoiding mistargeting and aggregation. d. Chemical properties of TMs that may hinder their membrane insertion. Hydrophilic loops need to be translocated across the membrane from the cytosol, and polar residues within the TM may be incompatible with the hydrophobic membrane. Created in BioRender. https://BioRender.com/4gn2ixa.

Yet the bilayer is not a uniform environment. Together with the lipid headgroups, it is ∼40–45 Å thick and defined by a steep polarity gradient, from hydrophilic headgroups at the surface to a highly hydrophobic core.^9,16^ TM sequences often mirror this gradient to optimize membrane compatibility (Figure 1b). For example, hydrophobic residues in the center of the TM contribute more strongly to insertion than those near the edges.^10^ While hydrophobicity remains the dominant determinant, other sequence features modulate insertion. Aromatic residues are often enriched at the membrane interface, forming an “aromatic belt” that interacts with lipid headgroups.^17,18^ Proline residues, though hydrophobic, can disrupt helicity and reduce insertion efficiency.^10,19^ Positively charged residues behave in more complex ways: they may localize near the interface by snorkeling their charged groups toward the aqueous phase,^20,21^ and they play a key role in determining TM orientation through the ‘positive-inside’ rule.^10,11,14,22–27^ This rule reflects the empirical observation that TMs tend to orient such that the side with more flanking positive charges faces the cytosol, rather than the exoplasmic side of the membrane (Figure 1b). Intriguingly, though established many years ago, the mechanistic basis of the ‘positive-inside’ rule is still unclear. Possible explanations include the electrochemical gradient across the inner *E. coli* membrane, lipid asymmetry and electrostatic interactions between the ribosome or the insertion machinery with the inserted membrane protein.^28–35^

The contribution of individual amino acids to membrane insertion has been quantified in various systems, leading to the development of so-called hydrophobicity scales.^10,11,14,24–27^ These scales estimate the energetic cost or benefit of placing each amino acid at different positions along a TM segment. Despite being derived using different experimental and computational approaches, and in diverse membrane contexts, the resulting scales are quite consistent. Their profiles largely reflect how well each residue matches the physicochemical gradient of the membrane. Hydrophobicity scales are widely used to predict the location of TMs in protein sequences, and they perform surprisingly well, correctly identifying ∼80% of TM helices in membrane proteomes.^13,36^ Recent methods that take into account additional information increase this accuracy further.^37^ In this sense, the basic chemistry of insertion appears almost deceptively simple.

Yet in living cells, the situation is more complex. Correct membrane insertion competes with many other processes, and multiple points of failure can lead to misinsertion.

First, a eukaryotic cell contains several distinct membranes, and each membrane protein must insert into its correct target. While some MPs localize to more than one organelle, most are restricted to a specific compartment, and mistargeting can compromise fitness or cause toxicity^38,39^ (Figure 1c). The lipid composition varies between these membranes and the resulting biophysical properties of membranes influence insertion in ways that are only beginning to be understood.^9,40^ For example, tightly packed lipid tails may hinder spontaneous insertion, while loosely packed headgroups can facilitate insertion, likely by easing the initial access of the polypeptide to the bilayer’s hydrophobic core.^41–43^ However, how such lipid-related phenomena shape insertion across the different cellular membranes remains poorly defined.

In parallel, the TM segments of MPs vary widely in their compatibility with the membrane. While many TMs are well-matched to the bilayer, others are not—especially in multispanning MPs, where TMs often contain polar or charged residues, or are shorter than the membrane’s hydrophobic core.^10,20^ In the folded protein, such features are typically buried within the protein interior and shielded from the lipid environment. But during insertion, before folding has occurred, this protective environment is absent, making these TMs suboptimal for insertion at that stage (Figure 1d).

Even under more favorable conditions, TM insertion competes with other cellular challenges. The very property that promotes insertion, TM hydrophobicity, also makes these segments prone to aggregation in the aqueous cytosol (Figure 1c). To avoid aggregation and misfolding, TMs must be inserted rapidly and efficiently.^44,45^ An additional obstacle stems from the structure of membrane proteins themselves: TMs are typically flanked on both sides by hydrophilic segments—either loops connecting adjacent TMs, or terminal tails.^3^ The loops and tails that end up on the exoplasmic (i.e., noncytosolic) side must cross the membrane during insertion. While short, uncharged exoplasmic loops or tails might passively diffuse across the bilayer, most are too hydrophilic to do so unassisted on a biologically relevant time scale^46^ (Figure 1d). Thus, while spontaneous insertion is possible and has been observed *in vitro,* ^47–50^ where compounding processes are fewer, in cells, it would be inefficient and error-prone. Cells overcome these challenges with specialized machineries that coordinate, accelerate, and proofread the insertion process, ensuring a functional and properly folded membrane proteome.

## 3 The MP Biogenesis Toolkit: The Cellular Machinery for Targeting and Insertion

### 3.1 Targeting MPs to Their Destination Membrane

The majority of membrane proteins are synthesized by cytosolic ribosomes and must reach the correct membrane before they can insert into it. *In vitro*, TMs are sufficiently hydrophobic to associate with membranes spontaneously.^48^ In cells, however, the crowded cytosol poses a risk of aggregation before the TM reaches its destination. This challenge is compounded in eukaryotes, which contain multiple membrane-bound organelles. Without dedicated guidance, MPs may mislocalize to the wrong membranes,^51^ where they risk misfolding, loss of function, degradation, or toxic effects.

To overcome these hurdles, cells rely on dedicated targeting pathways. While some targeting can already occur on the level of the mRNA,^52–56^ the newly synthesized nascent protein is typically the targeted component. Targeting pathways consist of soluble factors that recognize targeting signals within the sequence of the newly synthesized protein and guide it through the cytosol to the correct membrane.^4,57,58^ Targeting signals fall into two main categories: noncleavable elements that are part of the mature protein, typically TMs and their flanking regions,^59^ and cleavable signals, such as hydrophobic signal peptides located at the N-terminus of the protein. Signal peptides, which also direct secreted proteins to the membrane, are promptly removed after targeting, leaving no trace in the mature protein.^4,60^ In the final step of the targeting cascade, the client protein is handed off to the membrane insertion machinery, typically a translocon or insertase, which mediates its insertion into the lipid bilayer.^38,57^

In eukaryotic cells, multiple such pathways coexist to serve distinct organelles. Even in bacteria, which contain only a single cytosol-facing membrane, at least two parallel pathways operate to direct different classes of membrane and secreted protein clients to the plasma membrane.^60–64^ Several recent reviews comprehensively cover the complexity of protein targeting across organelles and membrane systems. Here, we limit our focus to a few core principles.

The best-characterized targeting pathway is the Signal Recognition Particle (SRP) system, conserved from bacteria to humans. This pathway is covered in detail in several comprehensive reviews.^4,38,65^ SRP scans ribosomes during translation and binds the first N-terminal TM or cleavable signal peptide that emerges out of the ribosome.^63,66–68^ It then targets the ribosome-nascent chain complexes bearing these signals to the SRP receptor at the membrane, allowing handover to insertion machineries such as the Sec translocon at the eukaryotic endoplasmic reticulum (ER) or bacterial plasma membrane^65,69–72^ (Figure 2a). SRP thus provides dual protection against aggregation: it shields hydrophobic TMs from the aqueous environment^59^ and ensures that *subsequent TMs* that follow the N-terminal signal are synthesized adjacent to the insertion machinery. Indeed, many ribosomes synthesizing membrane proteins are already targeted and docked on translocons,^73^ enabling *cotranslational insertion* of TMs as they emerge, thereby minimizing their exposure to the cytosol (Figure 2b).

**Figure 2:**
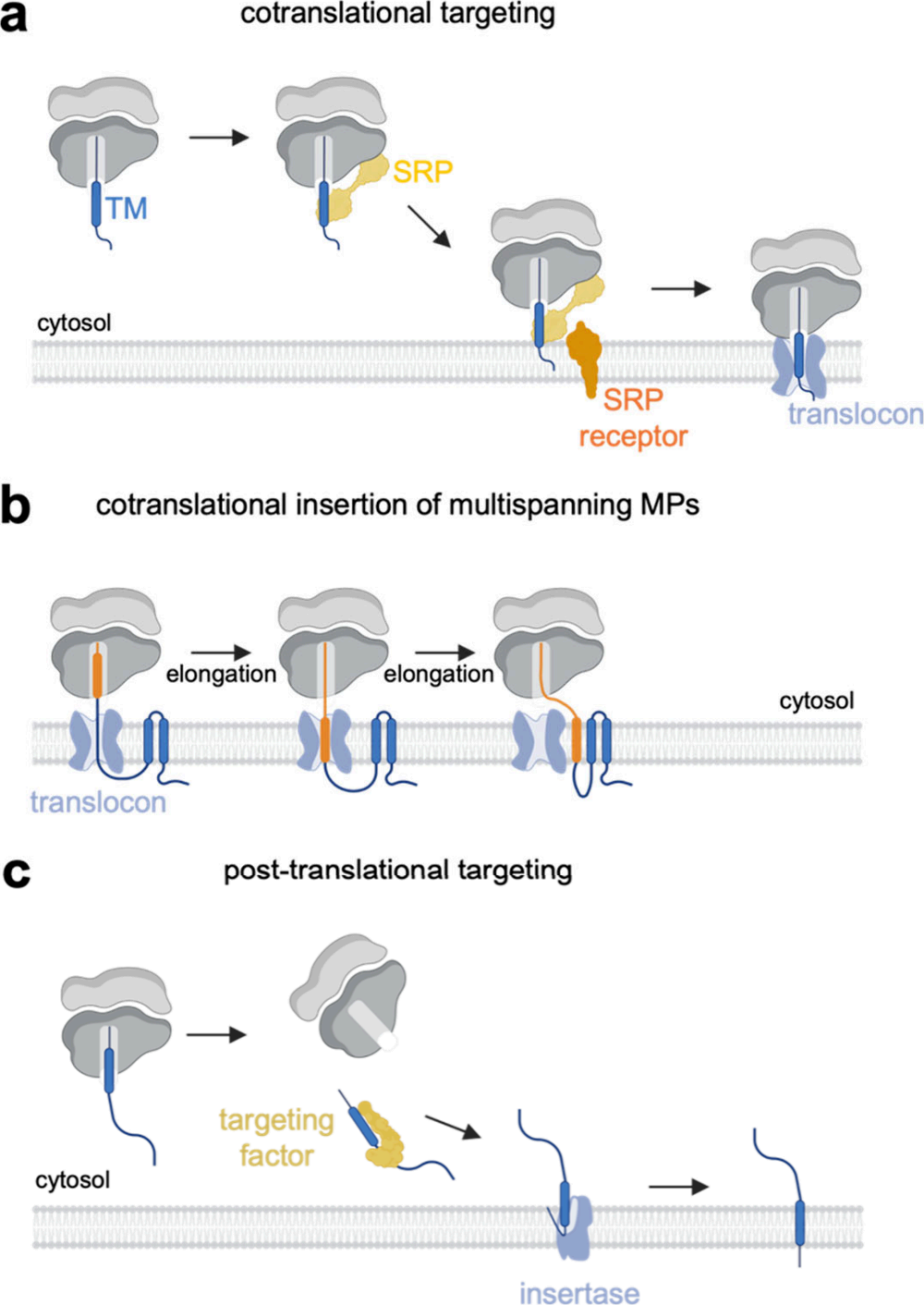
**Simplified schemes depicting core principles of MP targeting.** a. Cotranslational targeting, with the SRP pathway as an example. The targeting factor SRP binds the TM as soon as it emerges from the ribosome. With the help of the membrane-bound SRP receptor, the ribosome–nascent chain complex is delivered to the membrane insertion machinery, typically the Sec translocon. b. Cotranslational insertion of multispanning membrane proteins. The ribosome-nascent chain complex docks onto a membrane translocon. As translation progresses, the newly synthesized TM can insert into the membrane immediately upon emerging from the ribosome. c. Post-translational targeting of TA proteins. Because the TM remains buried in the ribosome exit tunnel and is inaccessible to targeting factors, translation must terminate before it can be recognized. Once the ribosome dissociates, the exposed TM is captured post-translationally and delivered to the appropriate membrane insertase. Created in BioRender. https://BioRender.com/agdmbo0.

However, not all clients can use the cotranslational pathway. Tail-anchored proteins, for instance, contain a single C-terminal TM that is synthesized too late for SRP to engage the ribosome.^74^ This is because the TM is positioned near the protein’s C-terminus and only emerges from the ribosome once translation is complete. Prior to that, it remains buried within the ribosomal exit tunnel, which can accommodate ∼30–40 amino acids and is inaccessible for efficient SRP binding.^75^ As a result, by the time the hydrophobic TM is fully exposed, the protein has already dissociated from the ribosome, precluding cotranslational targeting (Figure 2c). These proteins therefore require post-translational targeting. In bacteria, SRP can also act post-translationally;^62,76^ in eukaryotes, dedicated pathways evolved, including the GET and EMC pathways.^74,77–79^ In both, the hydrophobic tail anchor is chaperoned in the cytosol and delivered to a membrane insertase (Figure 2c).

SRP also cannot target proteins destined for other organelles, such as mitochondria, chloroplasts, or peroxisomes.^57,80,81^ These rely on alternative pathways,^57,80,81^ which were initially considered post-translational but are now recognized as partly cotranslational. Mitochondria, for example, import most of their proteins from the cytosol, using several distinct pathways tailored to different classes of proteins. Outer membrane proteins engage various cytosolic chaperones and targeting factors, depending on their topology,^82,83^ and are then inserted into the outer membrane via dedicated insertion machineries.^84,85^ In contrast, proteins destined for the inner mitochondrial membrane typically bear an N-terminal presequence that directs them across the outer membrane via the TOM complex, followed by insertion through inner membrane machineries such as TIM23, TIM22, and the Oxa1 insertase.^86,87^ In addition, a subset of inner membrane proteins are synthesized within mitochondria by mitochondrial ribosomes and inserted cotranslationally by the Oxa1 insertase. Thus, mitochondrial proteins are highly diverse in their origin, topology, and targeting signals, and are accordingly matched by a diverse set of targeting and insertion pathways.

A central puzzle is how targeting specificity is achieved, especially as most pathways recognize similar features - hydrophobic TMs. Specificity arises from subtle sequence differences in the TM and flanking regions, which are read and interpreted by the targeting and insertion machinery.^59,66,88^ Importantly, each component in a pathway can contribute to sharpening this specificity: initial recognition, chaperoning, and membrane delivery steps all act as selective filters.^38,89,90^ Targeting factors compete in the cytosol,^38,51^ and small differences in client sequences or exposure timing can shift the balance of affinity and capture efficiency toward one pathway over another. Some overlap between pathways exists, especially in the ER, where multiple routes can handle a partially overlapping set of clients.^4,51,74^ Yet this redundancy also provides robustness, allowing alternative pathways to backup one another. Notably, targeting pathways are not necessarily committed to one insertion machinery. For example, the SRP in both eukaryotes and prokaryotes has been shown to target Oxa1-family insertases, in addition to the Sec translocon.^31,91^ Finally, insertion machineries may contribute to specificity by rejecting incompatible clients. For instance, the ER-resident EMC does not efficiently handle mitochondrial tail-anchored proteins, in part due to their positively charged flanking residues.^88,89^

While no single pathway ensures perfect fidelity, their combination, together with cytosolic chaperones and organelle-specific insertases, provides robust sorting. Mistargeting can still occur, but surveillance systems exist to degrade or redirect mislocalized proteins (see section 5 below). Thus, targeting defines the subset of membrane proteins that arrive at a given membrane, where they can be inserted co- or post-translationally by the local machinery.

### 3.2 The Core Insertion Machinery: Translocons and Insertases

Once targeted to the correct membrane, the TMs of client MPs are inserted through specialized machineries. These systems evolved to capture the TM from the targeting pathway or ribosome, insert it into the bilayer, and translocate flanking hydrophilic loops or tails across the membrane in a precise and kinetically efficient manner. The two best understood machineries belong to two ubiquitous families with distinct architectures: the Sec translocon and the Oxa1-family insertases. Sec translocons contain a protein-conducting pore, whereas Oxa1-family insertases form a membrane-embedded hydrophilic groove that can only translocate short polar regions of a substrate across the bilayer during insertion. Sec translocons and Oxa1-family insertases handle TMs at the primary sites of MP insertion—the cell membrane in prokaryotes and the ER in eukaryotes. Additional machineries exist in other organelles such as mitochondria, peroxisomes, and chloroplasts, which are covered in dedicated reviews.^86,92–101^ Here, we focus on the Sec translocon and the Oxa1 family insertases.

#### 3.2.1 The Sec Translocon

The Sec translocon is the best understood of all insertion systems. First identified more than 40 years ago,^102–104^ it has since been characterized extensively by structural and biochemical approaches, for reviews, see.^5,31,100,105^ In bacteria it comprises SecY, SecE, and SecG, together termed SecYEG, while in eukaryotes the homologous Sec61 complex comprises Sec61α, Sec61γ, and Sec61β. Two small subunits (SecE/G or Sec61γ/β) are peripheral and stabilize the assembly, whereas the SecY/Sec61α, with its 10 TMs, provides the central channel.

The Sec translocon typically acts cotranslationally during membrane protein biogenesis, inserting TMs directly from the ribosome into the membrane. It can also function post-translationally, most clearly for soluble secretory precursors.^105,106^ Three structural features define its function:

First, docking of the translocon at the exit tunnel of the ribosome through interactions of cytosolic loops of SecY/Sec61α allows nascent chains to pass directly from the ribosome exit tunnel into the translocon, minimizing cytosolic exposure.^105,107,108^ A small gap between the ribosome and the channel permits the cytosolic loops of MPs to emerge in the cytosol.^109^

Second, SecY/Sec61α contains a protein-conducting channel, an hourglass-shaped pore traversing all the way from the cytosol to the exoplasmic side of the membrane.^110^ The channel can translocate any unfolded polypeptide across the membrane, even very hydrophilic or long sequences (Figure 3a). This channel mediates most of the secretion of large hydrophilic domains, whether they are exoplasmic loops of MPs or entire secretory proteins that follow a cleavable signal peptide.^6,111^ A small plug and pore-ring residues seal the permeability barrier in the resting state and during polypeptide translocation.^112–115^

**Figure 3:**
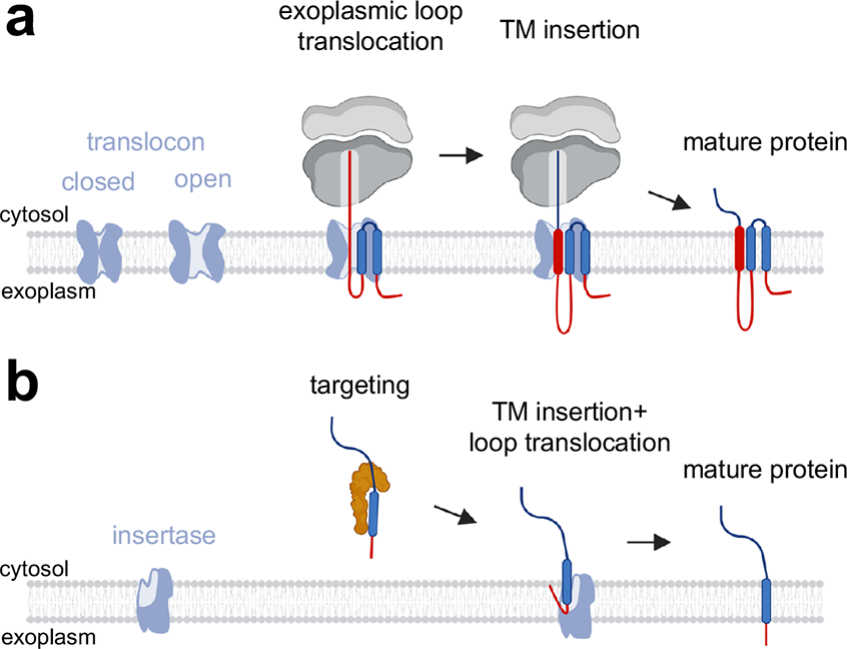
**Mechanism of TM insertion and exoplasmic loop translocation by the Sec translocon and insertases.** a. The Sec translocon can translocate long exoplasmic loops through its hydrophilic transmembrane pore. TMs are inserted via the lateral gate. b. Insertases insert TMs and translocate their flanking exoplasmic loops simultaneously, through a shallow hydrophilic groove. Created in BioRender. https://BioRender.com/xcfc94v.

The third crucial feature is the lateral gate, which is the major site of TM insertion. This gate is situated between the pseudosymmetric halves of SecY/Sec61α, which form a clamshell-like structure. To allow insertion of hydrophobic sequences into the bilayer, the two halves of the clamshell can move apart, forming a gate that opens laterally to the membrane, thus exposing the pore of SecY/Sec61α to the phospholipid bilayer and allowing insertion^112,113,116,117^ (Figure 3a).

The prevailing “lateral gate” model of insertion, established mainly from single-TM and signal peptide substrates, remains the consensus view of Sec-mediated insertion (for current reviews see^4,5,31,100^ ). In this model, binding of a hydrophobic signal peptide or TM induces gate opening, allowing the TM to integrate in a transmembrane configuration at the lateral gate. At the same time, the plug is displaced, allowing hydrophilic loops to translocate through the Sec pore while an additional TM may partition laterally into the bilayer through the lateral gate.^112,113,116,117^ While this mechanism was once viewed as the dominant route for TM insertion, it is now clear that insertases contribute substantially as well.

#### 3.2.2 Oxa1-Family Insertases

The second major class of insertion systems is the Oxa1 family of insertases. First discovered in mitochondria (Oxa1 and its homologue Oxa2/Cox18), the family also includes the chloroplast insertases Alb3 and Alb4, and the bacterial member YidC, all of which mediate MP insertion.^118–126^ Oxa1-family insertases can act either co- or post-translationally.

The bacterial insertase YidC is the best studied example. It facilitates TM insertion both co- and post-translationally, functioning independently or in conjunction with the Sec translocon complex^105,127^ (see below). YidC contains a ribosome-binding site formed primarily of a positively charged cytosolic loop and the C-terminus,^128^ yet it can also capture TMs outside the ribosomal context. A cytosolic coiled-coil in its first loop contributes to substrate capture, ensuring handover of hydrophobic segments from either the ribosome or SRP into the membrane^128–130^ (for in-depth reviews, see the literature^31,100,131–133^ ).

Unlike Sec, the conserved TM core of YidC does not form a continuous channel across the bilayer. Instead, it creates a shallow hydrophilic groove that extends halfway into the membrane.^134–137^ This vestibule accommodates short hydrophilic stretches flanking the TM of the client protein, facilitating their translocation, while adjacent hydrophobic surfaces guide TM segments into the lipid phase (Figure 3b). Molecular simulations and cysteine-scanning studies further show that YidC induces local membrane thinning, effectively lowering the barrier for polar groups to cross.^136–138^ Together, the groove and membrane thinning create a permissive environment for inserting helices flanked by short polar regions.^139^ However, the absence of a pore limits the range of substrates: insertases cannot translocate long hydrophilic loops or domains, distinguishing their specialization from that of the Sec translocon.

The eukaryotic ER possesses three distant homologues of the Oxa1 family: Get1/WRB, EMC3 and TMCO1. While YidC is a single-subunit insertase, in the ER homologues, the insertase architecture is effectively split across two interacting proteins. Get1/WRB, EMC3 and TMCO1 provide the YidC-like three TM conserved core, while Get2/CAML, EMC6 and OPT1 (C20orf24) pack against it to complete and stabilize the functional insertase unit.^6,127,140^ The essential architecture is preserved in all homologues: a three-TM insertase, a cytosolic coiled-coil, and a hydrophilic groove that acts together with local membrane thinning.^141–147^ The expanded eukaryotic insertase repertoire might reflect the need to accommodate a broader range of substrates under the stronger quality control demands of a compartmentalized cell. In eukaryotic cells, the GET complex (Get1/WRB with Get2/CAML) inserts tail-anchored proteins,^78,127^ while TMCO1/OPT1 constitutes the metazoan-specific GEL complex, recently implicated in multispanning MP insertion.^33,140,143,148^ EMC3/6 form the heart of the larger EMC complex that is composed of eight subunits in yeast and nine in humans, and handles a subset of tail-anchored and multispanning MPs.^6,74,77,149–151^ EMC has emerged as a central hub in membrane protein biogenesis, combining a transmembrane insertase core with multiple peripheral modules involved in client recognition and lipid remodeling - functions that are only beginning to be understood.^77,144,149,150,152–155^

#### 3.2.3 Functional Parallels between Translocons and Insertases

Despite their structural and mechanistic differences, the Sec translocon and Oxa1-family insertases share some functional similarities. The initial engagement of a signal peptide or signal anchor at the lateral gate, prior to full pore opening, is conceptually reminiscent of Oxa1-family insertase function.^3,156^ Moreover, both machineries, which are the first to encounter nascent TMs in the membrane, can function as intramembrane chaperones that stabilize unstable helices until the protein is fully inserted and folded. Hydrophilic TMs in particular often remain associated with the Sec translocon until later steps of insertion or until other domains arrive to promote folding.^157–161^ A recent cryo-EM structure of a multispanning MP inserting via a SecA-stabilized SecY complex suggests additional chaperone-like roles on both sides of the membrane, with unfolding promoted cytosolically and folding promoted extracytosolically.^162^ Multiple lines of evidence indicate that Oxa1-family insertases share this chaperone function. For example, the bacterial YidC assists the folding of MPs such as LacY and MelB even independently of insertion.^163–166^ In the ER, the EMC has emerged as an intramembrane chaperone that can transiently engage misfolded proteins on their way to folding or assist subunit assembly (for more details see section 4.3).^144,152,154,155^ A recurring theme is that hydrophilic TMs are enriched among these client proteins. Their mismatch with the surrounding bilayer likely prevents free diffusion, explaining why these TMs remain associated with the insertion machinery.

Another emerging shared feature of insertion systems is their link to lipid remodeling. The bilayer thinning induced by Oxa1-family insertases is reminiscent of lipid scramblases, which use shallow hydrophilic grooves to facilitate lipid movement between leaflets.^167^ Indeed, recent studies suggest that insertases themselves may possess lipid-scrambling activity,^168^ and a similar activity has been proposed for Sec61.^168,169^ Since SecY was also shown to locally thin the bilayer,^162^ lipid scrambling may represent a general principle of insertion machineries: transiently softening the bilayer to allow the passage of hydrophilic residues, while directing hydrophobic helices into the membrane.^168^

### 3.3 Auxiliary Partners of the Insertion Machinery

The cellular machinery for protein insertion and translocation handles an extremely diverse array of secreted and membrane proteins. To efficiently accommodate this vast repertoire of substrates, translocons and insertases can cooperate and employ a dynamic network of auxiliary proteins and protein complexes. While these factors are varied between bacteria and eukaryotes, they perform similar functions, aiding in TM insertion, exoplasmic loop translocation, and chaperoning the immature protein segments in and out of the membrane.

#### 3.3.1 Bacterial Auxiliary Factors

In bacteria, this network centers around the SecYEG translocon, as only a few substrates can insert with YidC alone. SecYEG can be found in complexes with YidC, the essential ATPase SecA, the auxiliary SecDF complex (which in *E. coli* also contains the YajC protein), or as part of the holotranslocon, which includes SecDF­(YajC) and YidC (for comprehensive reviews, see the literature^105,131,170^ ).

SecA is the best-characterized auxiliary factor associated with the bacterial translocon. It is a cytosolic ATPase that drives the translocation of large hydrophilic segments, typically secretory proteins or periplasmic loops of membrane proteins, across the membrane. SecA primarily acts post-translationally on secreted proteins bearing cleavable signal peptides, playing a dual role in their targeting and translocation. It recognizes the signal peptide, delivers the precursor to the SecYEG channel,^105,170,171^ and then uses ATP hydrolysis to drive conformational cycles that open the channel and push the substrate across.^105,172^ This activity is further enhanced by the proton-motive force.^64,173^ Although best known for post-translational secretion, SecA can also function cotranslationally. It associates with ribosome–nascent chain complexes translating secretory proteins with highly hydrophobic signal peptides, as well as with certain integral membrane proteins that contain large exoplasmic (i.e., periplasmic) loops.^64,105,174^ In these cases, SecA likely assists the SecYEG channel in threading extended polypeptide segments across the bilayer while translation proceeds.^64,105,174^

SecDF is another auxiliary complex, yet its function is less clear. While SecA facilitates substrate movement through SecYEG on the cytoplasmic side, the auxiliary SecDF complex may assist on the periplasmic side. A model based on structural and functional data suggests that SecDF binds substrates as they emerge from SecYEG, and couples proton transport with large conformational changes to pull substrates through.^105,175–178^

SecYEG also cooperates with the YidC insertase, either as a heterotetrameric complex, or as part of the holotranslocon.^179,180^ The relative abundance, substrate repertoire, and roles of these YidC-containing complexes are not well resolved. YidC was suggested to aid SecYEG in the insertion of multispanning proteins, the assembly of membrane oligomeric complexes, and to act as a folding chaperone^105,163,181–183^ (see also below).

Finally, interactions between the holotranslocon complex and the BAM complex (β-barrel assembly machinery), along with periplasmic chaperones like Skp, SurA, and the YfgM-PpiM complex, are crucial for the biogenesis of outer membrane proteins, but are outside the scope of this review. For current reviews see.^105,172,184,185^

#### 3.3.2 Eukaryotic Auxiliary Complexes

The greater diversity of substrates and membrane compartments in eukaryotes is reflected in the complexity of the ER insertion and translocation machinery. The core Sec61 translocon operates within a network of protein complexes. Some, such as the Sec62/63 or TRAP complexes, assist in insertion and translocation of proteins by opening or stabilizing the lateral gate or the pore of the Sec translocon. Others, like the ‘multipass translocon’ (MPT) complexes BOS (back of Sec61), GEL (GET- and EMC-like), and PAT (protein associated with translocon), cooperate with the Sec translocon to allow the efficient insertion of multispanning proteins. The translocon also associates with modifying enzymes like the oligosaccharyltransferase complex (OST), which may stabilize the protein topology by adding a large hydrophilic group to a luminal loop, and the signal peptidase complex, which cleaves signal peptides.^100,106,109,186,187,77,144,149,150,152–155^ Though our understanding of the mechanistic details is incomplete, efficient insertion of different substrates is thought to require distinct subsets of this intricate auxiliary network. For example, the Sec62/63 complex is mostly involved in post-translational translocation of secretory proteins, while the heterotetrameric TRAP complex (translocon-associated protein complex) is found in over 90% of metazoan ER ribosome-translocon complexes.^73,106,188^ Both complexes were suggested to assist in the insertion and translocation of “difficult” substrates.

TRAP was suggested to assist in the translocation of signal peptide-containing precursors and multispanning proteins with weaker lateral gating activity by stabilizing the open Sec61 conformation.^116,189–192^ It also interacts with the ribosome on the cytoplasmic side and with the OST complex on the lumenal side, enhancing N-glycosylation of a subset of proteins.^106,193^ Similarly, the Sec62/63 complex (that in yeast includes also the nonessential Sec71/72 subunits) was shown to open the lateral gate and help the channel handle signal peptides or TMs with lower hydrophobicity.^106,188,190,194–197^ Sec63, through its J-domain, also recruits and activates the lumenal HSP70-family chaperone BiP (Kar2 in yeast). Similar to the bacterial SecA, BiP utilizes ATP to enhance translocation across the membrane. However, while the SecA motor pushes the polypeptide chain from the cytosolic side, the BiP chaperone binds the emerging polypeptide in the ER lumen in an ATP-dependent manner and functions as a molecular ratchet that blocks back-sliding into the cytosol.^105,111,198,199^ Additionally, BiP binding to a luminal loop of Sec61α limits ER Ca^2+^ leakage through the pore.^106,200–202^

Other less-characterized accessory factors include TRAM1 (translocating chain-associated membrane protein), RAMP4 (ribosome-associated membrane protein 4, also known as SERP1 in mammals and Ysy6 in yeast) and the metazoan-specific prolyl isomerase FKBP11. TRAM1 cross-links to signal peptides and TMs during their translocation, and its depletion affects translocation efficiency of some signal peptides *in vitro* and decreases the expression of several secretory and membrane proteins *in vivo.* ^106,190,203^ RAMP4 cross-links to nascent chains during their translocation through Sec61.^106,188,204,205^ Recently, it was identified as a common component of ribosome-translocon complexes, and based on the structural details was suggested to act as a surrogate signal peptide, facilitating the transport of certain sequences through Sec61 channel during later translocation stages.^116^ FKBP11 is an ER-localized prolyl-isomerase and the only member of the family that has a TM. It was shown to bind ribosome-translocon complexes during translation of secretory and membrane proteins, with a preference for proteins whose first translocated segment is long. As FKBP11 depletion causes destabilization of such substrates, it was suggested to function as a translocon accessory factor. However, whether this function requires prolyl isomerization is still unknown^206^

A recently identified specialized ribosome–translocon assembly, the MPT, contains three additional complexes, BOS, GEL and PAT, that bind to the back side of Sec61 and form a dynamic, modular complex that mediates the insertion of multispanning membrane proteins.^33,73,140,143^ (for more details see section 4.2).

While some ribosome–Sec61 complexes assemble as MPT, most other assemblies form ‘secretory translocons’, which contain Sec61, TRAP, and the oligosaccharyltransferase complex A (OSTA).^73^ These translocons operate with an open Sec61 pore to translocate long secreted segments or large exoplasmic loops, whereas MPT complexes, built around closed Sec61 channels, mediate the integration of TMs connected by short translocated loops. The two architectures are thought to represent mutually exclusive states of the ribosome–Sec61 assembly, tuned to different needs of nascent chains. This division of labor allows the ribosome–Sec61 platform to flexibly accommodate the diverse topologies of complex membrane proteins.^207^ Intriguingly, there are differences in the auxiliary complexes between different eukaryotic organisms. Not all of TRAP subunits are evolutionarily conserved in plants or yeast,^116^ and its role in nonmetazoan eukaryotes is not clear. The mammalian Sec62 acquired a ribosome-binding domain, and the complex was also demonstrated to participate in the cotranslational insertion of several proteins.^106,208^ Fungi and other eukaryotic lineages lack GEL and BOS homologues, but a recent cryo-EM structure suggests the existence of an alternative fungal MPT complex that consists of SEC61, TRAPα, the PAT complex and SND3 (SRP-independent 3), a novel fold insertase.^209^

Together with the variability observed in bacterial translocon assemblies, these differences underscore an evolutionary theme of modular adaptation. Across evolution, diverse combinations of auxiliary factors have evolved to facilitate efficient and accurate insertion of nascent membrane proteins.

## 4 Inserting the Diverse Membrane Proteome: Co-adaptation between TM Sequences and Insertion Routes

The membrane proteome is remarkably diverse, encompassing single-pass receptors, multispanning transporters, channels, and enzymes, each with distinct topologies and chemical features. Despite this variety, all must integrate efficiently into the correct membrane to function. This challenge is met by a set of versatile insertion machineries that evolved to accommodate substrates with widely differing hydrophobicities, topologies, and folding constraints. At the same time, membrane proteins have adapted their own sequences to the capabilities and limits of these systems. Features such as the positioning of transmembrane helices, their hydrophobicity, and the polarity of connecting loops have been tuned to ensure compatibility with the available insertion pathways. The interplay between substrate design and machinery adaptability underlies the robust biogenesis of an exceptionally heterogeneous membrane proteome.

### 4.1 Insertion of Single-Pass Membrane Proteins

The need for diverse insertion routes and how proteins evolved to utilize them is nicely exemplified by the insertion of proteins possessing a single TM. Single-pass (or single-spanning) proteins consist of a single hydrophobic TM flanked by N- and C-terminal tails. They can adopt two orientations in the membrane, depending on which tail remains in the cytosol: N-exoplasmic/C-cytosolic (N-exo, C-cyt) or N-cytosolic/C-exoplasmic (N-cyt, C-exo). The orientation is influenced by several factors, among others, the distribution of flanking positive charges, consistent with the ‘positive-inside’ rule, which preferentially places positively charged residues on the cytosolic side.^22,210,211^

Despite their relative simplicity, single-pass MPs are targeted and inserted by distinct mechanisms depending on their orientation, sequence, and the position of the TM along the sequence. A key determinant is the length and hydrophilicity of the exoplasmic tail, which must be translocated across the membrane, from the cytosol to the trans side.^6^ Insertases can only translocate relatively short, less hydrophilic regions—up to about ∼50 residues in mammals,^127,212^ and likely even shorter in bacteria.^213,214^ Thus, proteins with short exoplasmic tails are typically inserted by Oxa1-family insertases, independently of the Sec translocon pore. However, longer and more hydrophilic tails generally require the Sec translocon involvement^215,216^ (Figure 4).

**Figure 4:**
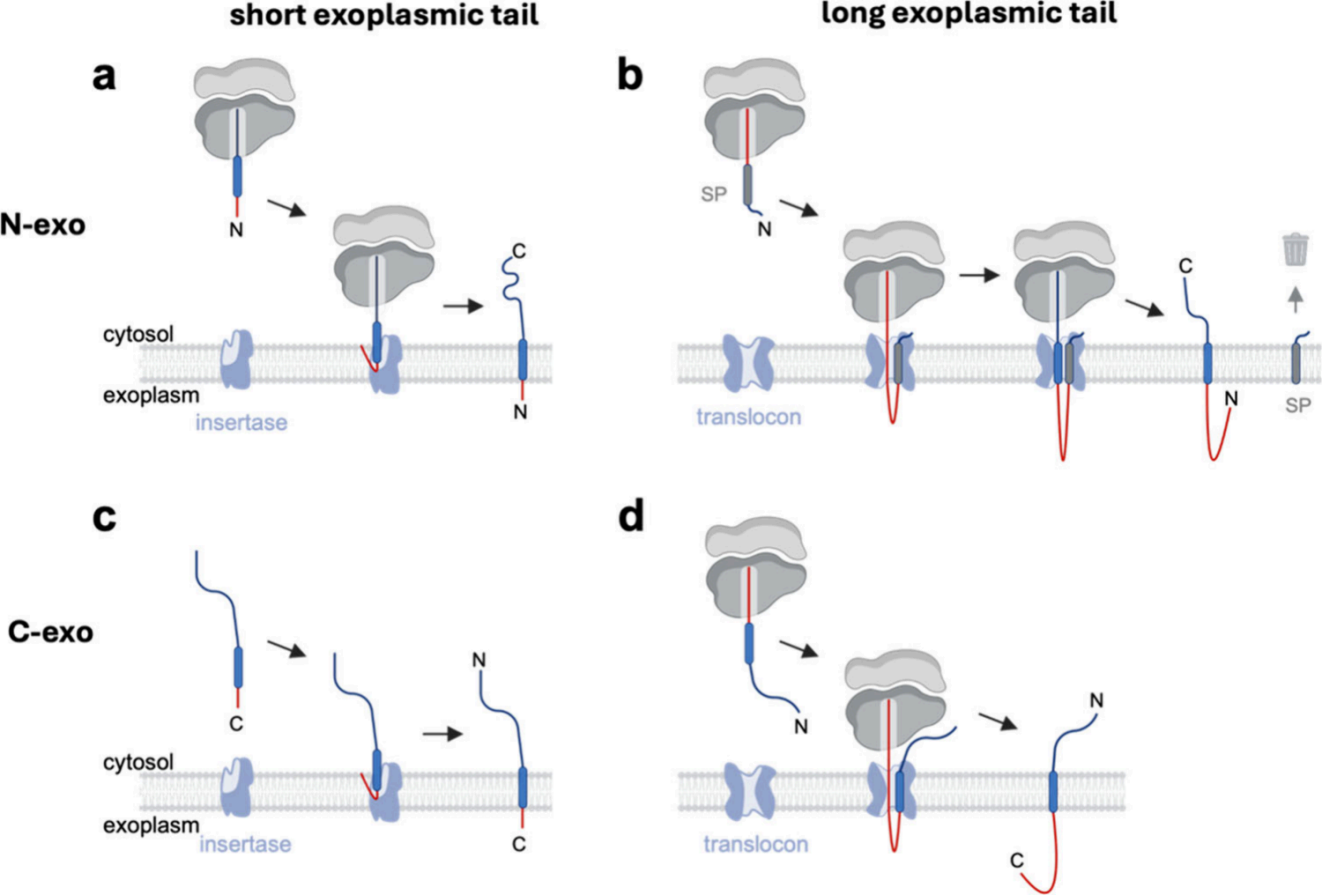
**Mechanism of insertion of single-spanning proteins.** The mechanism depends on the length (short/long) and orientation (N/C-exo) of the exoplasmic tail (red). SP in (b) indicates a cleavable signal peptide, which is necessary for the translocation of a long exolplasmic N-terminal tail. Created in BioRender. https://BioRender.com/na7rgtx.

For example, N-exo proteins with short N-terminal exoplasmic tails can insert efficiently via insertases like EMC in eukaryotes or YidC in bacteria, independently of the Sec translocon^124,149,217^ (Figure 4a). By contrast, proteins with longer N-exo tails require the Sec translocon and insert independently of insertases. These proteins evolved to carry an N-terminal cleavable signal peptide that targets them cotranslationally to the Sec translocon (Figure 4b). The signal peptide effectively acts as a transient TM, inserted into the membrane via the Sec lateral gate. Upon insertion, it opens the pore and recruits the secretory translocon auxiliary factors in eukaryotes, or SecA in bacteria. Once inserted with its C-terminus facing the exoplasmic side, the signal peptide directs the translocation of the following polypeptide through the Sec pore, much like a secreted protein. When the true TM arrives at the translocon, it integrates into the membrane through the lateral gate, halting further translocation and directing the C-tail to the cytosol. The signal peptide is then cleaved, leaving the mature exoplasmic N-tail.^4,218^

N-cyt/C-exo proteins follow a similar logic. For proteins with *long* C-terminal exoplasmic tails, the TM itself acts as a signal-anchor, which functions like a signal peptide but does not get cleaved. The TM is inserted by the Sec translocon and directs cotranslational translocation of the C-tail through the pore of the secretory translocon^91,219,220^ (Figure 4d).

C-exo proteins with a *short* C-terminal tail constitute a special case of well-studied proteins - tail-anchored (TA) proteins. As mentioned in section 3.1, these proteins undergo post-translational targeting and insertion. Their short C-exo tails allow them to be inserted by insertases without Sec involvement (Figure 4c). In *E. coli*, for example, TA proteins are thought to be inserted via the YidC insertase, with SRP handling their targeting post-translationally.^62,221,222^

In eukaryotes, TA proteins are more diverse and can target multiple membranes, making their biogenesis more complex. In the ER, TA insertion is mediated by two parallel pathways converging on two Oxa1-family insertase complexes: EMC and GET1/2.^77,78,223–226^ The choice between these pathways depends on TM hydrophobicity: highly hydrophobic TMs are routed through the GET pathway to the GET complex, while moderately or weakly hydrophobic TMs preferentially engage the EMC.^74,77,145,146^ Some TA proteins can use both pathways,^77,227^ and the less characterized SND pathway in yeast may provide an additional insertion route.^228^

TA proteins also insert into membranes other than the ER. For instance, in the mitochondrial outer membrane, TA proteins insert via recently identified machineries such as Mtch2 in humans or MIM1 in yeast, both distinct from the Oxa1 family.^84,85^ Their targeting specificity is determined by their sequence features, with mitochondrial TA proteins tending to have positively charged C-terminal regions and reduced TM hydrophobicity.^88,89,229–235^ While much of this specificity is imposed by soluble targeting factors, differences in the insertion machinery itself likely add an additional layer of selectivity.^89^

A final class, small MPs,^236^ consists of proteins under 100 amino acids, typically with a single TM. Due to their small size, they have modest extramembrane regions on both sides. They typically use post-translational targeting and insertion, but remain less well characterized in eukaryotes.^237,238^ In *E. coli*, some can be inserted by the YidC insertase alone,^239–244^ while others may use either YidC or SecYEG, with pathway choice influenced by features such as charge distribution in TMs or loops.^76,245,246^ Even single amino acid changes can switch both topology and pathway preference,^247^ highlighting a potentially blurred boundary between insertion routes in *E. coli*.

Altogether, the biogenesis of single-pass MPs illustrates how a moderate set of sequence features, tail length and sequence, polarity, TM hydrophobicity and position within the polypeptide, determine both orientation and pathway choice.

### 4.2 Insertion of Multispanning Membrane Proteins

Multispanning membrane proteins, which contain several TMs, account for most of the TMs in a typical proteome. These proteins face a multistep insertion process, with their first TM usually directing targeting to the membrane, while the remaining TMs are inserted once the ribosome is docked onto a translocon. The molecular details of these steps are better understood in eukaryotes.

The insertion of multispanning proteins starts like a single-pass protein carrying a long C-terminal extension that includes additional TMs. Accordingly, the insertion of the first TM follows the same logic as single-pass proteins. Multispanning proteins with an N-exo topology and a long translocated tail rely on a cleavable signal peptide and initially engage the secretory Sec translocon.^109,140,206^ In contrast, when the N-exo tail is short, the first TM can insert independently of Sec61 in eukaryotes and is insensitive to Sec61 inhibitors.^248,249^ Such TMs are thought to insert via the Oxa1-family insertase EMC, which was shown to support the insertion of the N-exo TM1 of GPCRs.^149,150,217^ If the first TM has a N-cyt topology, the secretory Sec61 translocon is required if the first translocated loop is long.^207^ Information about N-terminal TM insertion in prokaryotes is limited, but is expected to follow the same general rules.^109,124^

After the first TM inserts, the ribosome docks on the translocon and remains attached to it for most of the translation, maintaining continuous access to the membrane. This coordination allows the following helices to enter the bilayer as soon as they emerge from the ribosome, streamlining insertion with translation.^107^

Our understanding of how subsequent transmembrane helices insert has changed considerably in recent years. Earlier models proposed that these helices enter the membrane through the Sec translocon, one after another or sometimes in pairs, with the lateral gate mediating their insertion and the pore translocating the exoplasmic loops. This view was supported by extensive biochemical data, including *in vitro* reconstitutions showing that the Sec translocon alone can insert multispanning proteins^34,250^ (see also a recent review on the topic in this issue by Daniel J. Müller and colleagues). More recently, however, this model has been challenged, particularly in metazoans, as many multispanning proteins remain efficiently inserted even when the Sec61 lateral gate is inhibited. Biochemical, genetic, and structural analyses now point to the existence of a distinct ribosome-associated complex, the MPT, that engages these substrates.

The three accessory complexes of MPT, BOS, GEL and PAT, are preferentially recruited to a closed Sec61after insertion of the first TM,^4,33,73,109,140,143,251^ and form a lipid-filled cavity on the opposite side of Sec61’s lateral gate. Thus, insertion appears to occur at the ‘back side’ of Sec61 and does not require the lateral gate^33,140,143^ (Figure 5a). Although not directly shown, the Oxa1-family protein TMCO1, a component of the GEL complex, is thought to act as the insetase of the MPT.^4,6^ CCDC47, a component of the PAT complex, interferes with Sec61 opening and directs the nascent chain toward the lipid-filled cavity at the back of Sec61. Asterix, the other PAT component, chaperones partially hydrophilic TMs.^33,251^ The function of the BOS complex is unclear, but may relate to assembly or stability of the complex.^106^ Once assembled, the MPT is thought to facilitate insertion and provide a protected environment for the folding of multispanning proteins^33,143^ (Figure 5a).

**Figure 5:**
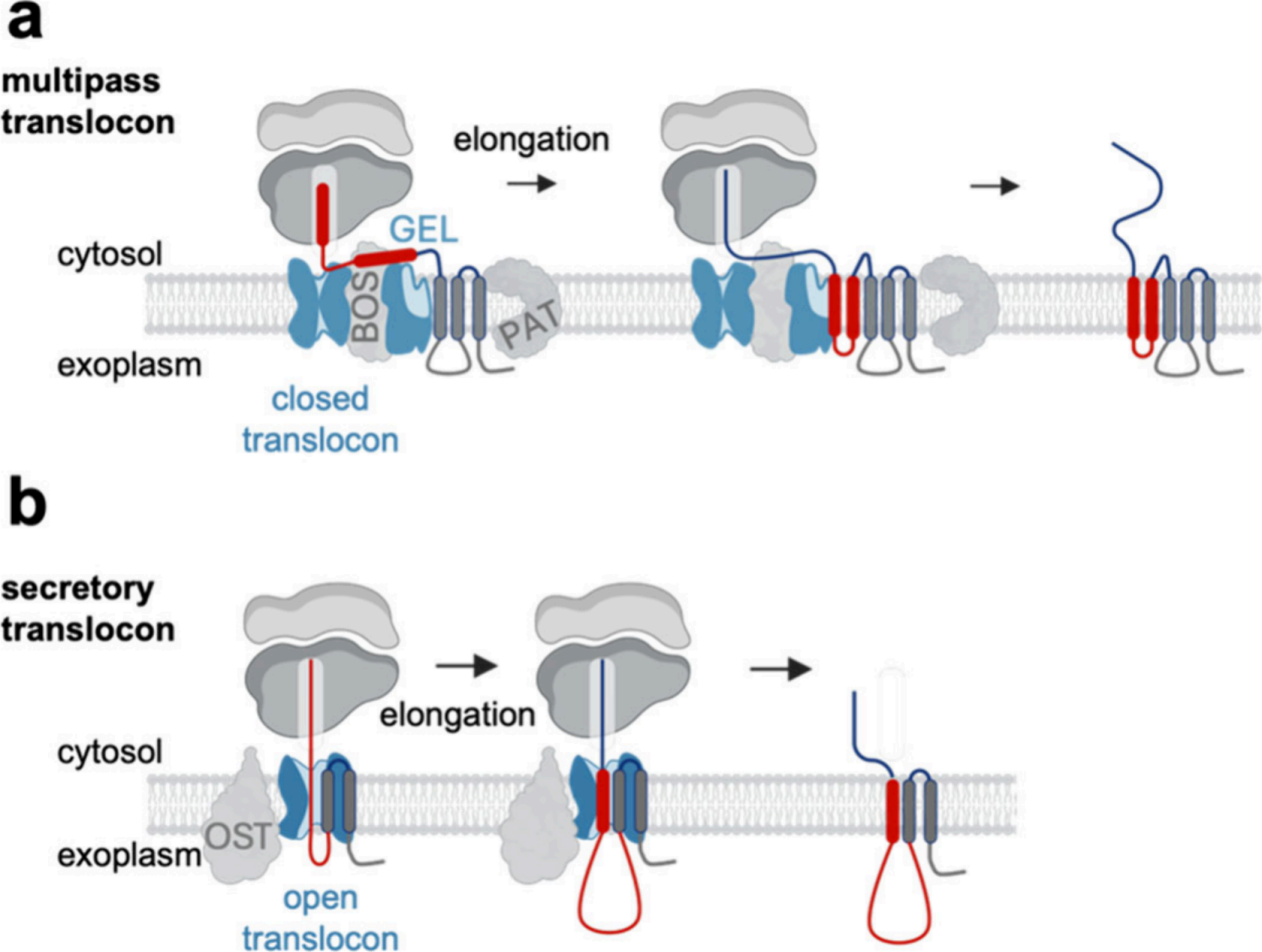
**Proposed mechanisms for the insertion of middle TM segments in metazoan multispanning proteins.** The inserted and translocated segments are shown in red, and the final protein topologies are shown to the right. (a) Insertion of two TMs connected by a short exoplasmic loop, indicated in red, by the multipass translocon. TM insertion and loop translocation occur at the backside of the translocon, where GEL (harboring a putative insertase), BOS, and PAT complexes reside. This route does not require opening of the Sec translocon. (b) Translocation of a long exoplasmic loop, followed by TM insertion, by the secretory translocon. This route depends on an open Sec translocon, which is associated with the glycosylation machinery (OST complex). Created in BioRender. https://BioRender.com/oha1t2y.

As the MPT employs an Oxa1 family insertase, it can only handle TMs linked by short exoplasmic loops, likely inserting them in pairs.^6,33,140^ Consistent with this limitation, most TMs of multispanning proteins have evolved compact topologies with short exoplasmic loops,^7^ suggesting adaptation to an insertion route that functions largely independent of the Sec61 pore.

Multispanning proteins that contain long translocated loops require access to the Sec61 pore, consistent with their sensitivity to Sec61 inhibitors.^33,207^ In such cases, the MPT disengages from the ribosome–nascent-chain complex and is transiently replaced by the secretory translocon, which includes OSTA and likely features an open channel^140,207^ (Figure 5b). The MPT also separates during translation of extended cytosolic loops.^207^ Together, these observations highlight the dynamic engagement of different translocon assemblies, with the sequence and topology of the nascent chain dictating which complex is recruited at each stage of insertion.

While better established in mammals, the multipass translocon is likely paralleled by analogous systems in bacteria and yeast. YidC, the Oxa1-family member in *E. coli*, can collaborate with the SecYEG translocon in the biogenesis of membrane proteins, and this complex is known to improve the insertion and folding of several multispanning proteins.^105,127,133^ Low-resolution structures and biochemical assays initially suggested that YidC is positioned with its hydrophilic groove facing the Sec lateral gate.^130,252–254^ However, a recent high-resolution cryo-EM structure of the SecYEG-YidC complex inserting a multispanning substrate showed that the TM was inserted without lateral gate opening. Reminiscent of the mammalian MPT, YidC was recruited to the back of SecY, presumably inserting the nascent chain through its hydrophilic groove.^255^

Similarly, though most Fungi lack GEL and BOS homologues, a recent cryo-EM structure describes an alternative MPT-like translocon, composed of Sec61, CCDC47 (and probably Asterix), TRAPα and SND3.^209^ This translocon complex is simpler in composition than the metazoan MPT and forms a smaller lipid-filled cavity in the membrane. In analogy to the metazoan MPT, Sec61β and CCDC47 work together in the SND3 translocon to prevent nascent chain access to the Sec61 channel. Moreover, SND3 has a hydrophilic groove within the cytoplasmic leaflet of the membrane and occupies the position of the TMCO1 insertase in metazoan MPT. These parallels point to a conserved architectural logic for multispanning insertion across kingdoms.

Notably, while improving MP biogenesis, the Sec translocon-insertase assemblies are not always essential for the insertion of multispanning proteins. TMCO1, the presumed insertase of the MPT, is dispensable for cell viability, indicating that other pathways can at least partially substitute for its function.^140,256^ Whether this redundancy reflects compensation by alternative insertases or the intrinsic flexibility of the Sec61 translocon, which may occasionally support insertion through its lateral gate, remains unclear. YidC, the sole Oxa1-insertase of *E. coli*, while essential for viability, appears dispensable for the expression and insertion of many multispanning MPs.^165,183^ Thus, the existence of multiple insertion routes in the membrane likely provides robustness.

Beyond the MPT, the EMC serves as another major insertase involved in the biogenesis of metazoan multispanning proteins. At least for some substrates, it engages SRP-targeted ribosomes early, before ribosome docking on Sec61. The nascent chain is then handed over to Sec61 where TMs (and presumably signal peptides) skipped by EMC can be inserted.^91^ EMC can also form a larger holoinsertase complex with the MPT components BOS, GEL and PAT, and these components might stabilize and chaperone less favorable N-exotails to facilitate insertion.^150^ Since steric constraints prevent EMC from binding to Sec61-bound ribosomes, the GEL/BOS/PAT complexes might also enable the handover of substrates to the MPT.^145,150^

Finally, not all transmembrane segments of multispanning proteins are inserted cotranslationally. The final helix can present a particular challenge, especially when followed by a short C-terminal tail.^161^ In such cases, the last TM remains within the ribosome exit tunnel when translation terminates, and the ribosome–translocon complex dissociates. Like tail-anchored proteins, these C-terminal TMs must therefore insert post-translationally. Recent studies indicate that this step is mediated by YidC in bacteria and by EMC in mammalian cells.^151,214^ Consistent with this, the C-terminal regions of such proteins tend to be short and moderately hydrophobic, features that likely evolved to favor efficient post-translational insertion through these Oxa1-family insertases.

Overall, the insertion of multispanning membrane proteins is a coordinated yet adaptable process. Distinct machineries such as the Sec translocon, the MPT, EMC, and the bacterial YidC engage the nascent protein in complementary stages to accommodate the varied topologies and chemistries of different substrates. Their engagement is guided by features of the nascent chain, including topology, loop length, and hydrophobicity. This modularity ensures that even complex multispanning proteins are efficiently integrated into the membrane.

### 4.3 Insertion of Polar or Weakly Hydrophobic TMs

The chemical features of a TM can challenge its insertion just as much as its position within the sequence. Efficient membrane insertion depends on the hydrophobicity of the TM. Yet, many TMs contain polar or charged residues that reduce their hydrophobicity. Such sequences are particularly common in multispanning proteins,^10^ such as ion channels and transporters, where polar residues are often essential for function. Polarity can sometimes be balanced by adjacent hydrophobic residues within the same TM,^257,258^ but this compensation is often incomplete (Figure 6a). As a result, TMs of only marginal hydrophobicity, which are predicted to insert inefficiently on their own, are frequent.^3,10,259^ In some cases, marginally hydrophobic TMs indeed fail to insert and may get translocated across the membrane. Such misinsertion events can invert the topology of downstream TMs, requiring substantial structural rearrangements to achieve the final functional topology.^260–264^ Although some of these errors can be corrected post-translationally (see Section 5 below), preventing them during insertion is likely advantageous. Both protein sequences and cellular machineries have therefore evolved to assist the integration of these challenging segments.

**Figure 6:**
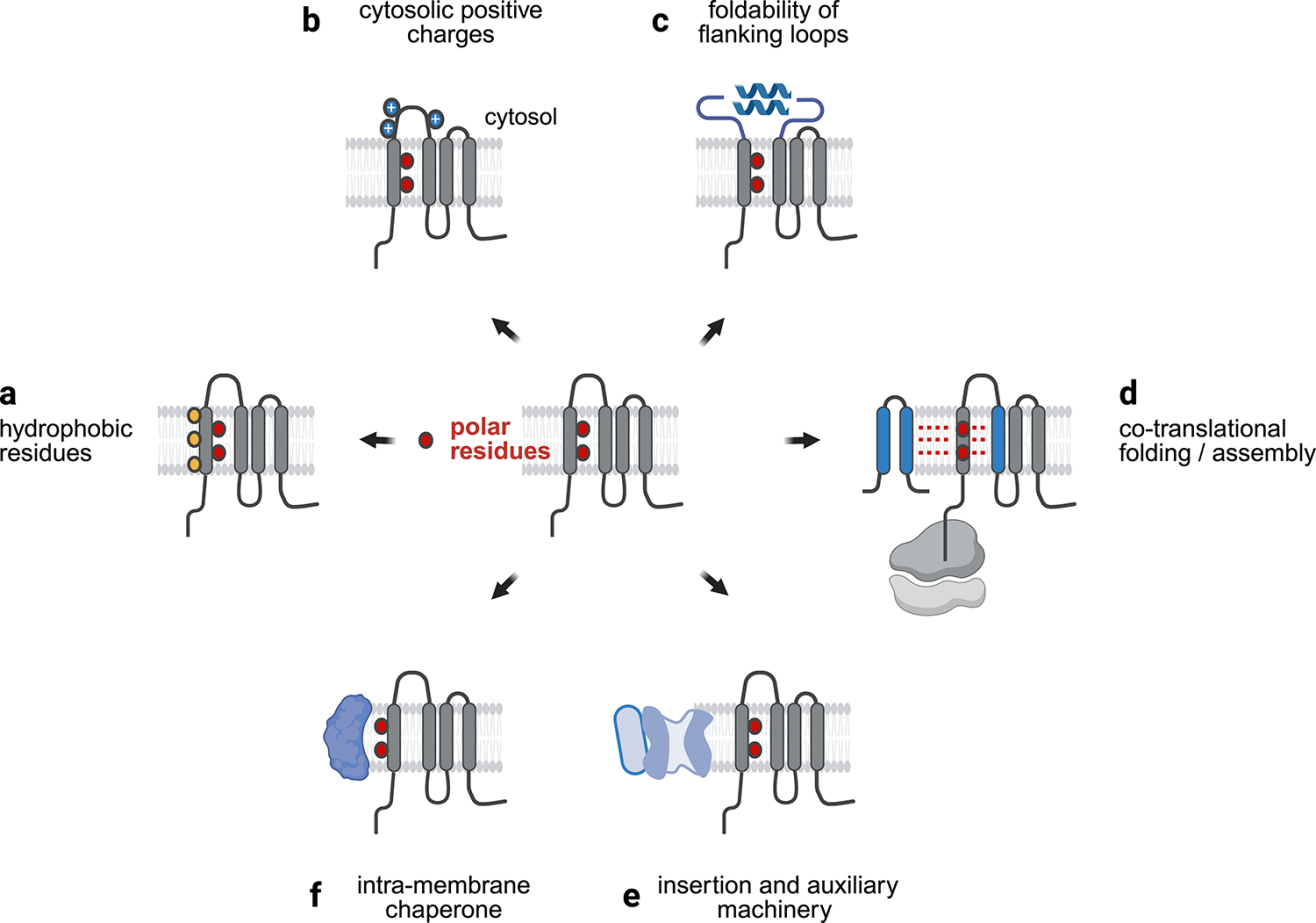
**Mechanisms promoting the insertion of polar or marginally hydrophobic TMs into the membrane.** Various factors can support insertion of TMs harboring polar residues, including: (a) Hydrophobic residues within the TM. (b) Cytosolic positive charges flanking the TM. (c) Foldability of loops flanking the TM. (d) Cotranslational interactions of the TM during folding or assembly. (e) Interactions with the insertion and auxiliary machinery, or (f) with intramembrane chaperones. Created in BioRender. https://BioRender.com/gixxzg3.

The most established strategy to promote insertion is through adaptive sequence context. Both downstream and upstream TM-flanking sequences have been shown to enhance the insertion of challenging TMs. While the precise mechanisms are not well resolved, several sequence features have been shown to contribute, including favorable flanking charges acting via the ‘positive-inside’ rule, the conformational flexibility or foldability of adjacent regions, and chaperone binding^10,265–269^ (Figure 6b,c).

Beyond linear sequence context, insertion can also be facilitated by the structural context of the TM. For example, adjacent TMs can aid insertion,^268–270^ potentially through direct interhelical interactions.^271–274^ These interactions can begin early, with helical hairpins forming already in the ribosomal exit tunnel or during insertion.^3,275,276^ Insertion may also depend on complex assembly,^46,277^ and oligomerization might already start cotranslationally^278^ (Figure 6d).

On the machinery side, since insertion is largely governed by the thermodynamic partitioning of helices into the lipid phase, insertion systems are not expected to efficiently drive highly polar transmembrane segments into the membrane. Translocons and insertases can lower the hydrophobicity threshold required for membrane entry, but these effects are modest^27,259,279^ (Figure 6e). Auxiliary factors such as Sec62/63 and TRAP may provide additional support (see section 3.3 above), although the molecular basis of their action remains unclear.

Once polar residues are inserted, however, they must be stabilized and shielded from the lipid environment. This likely involves intramembrane chaperones that can accommodate energetically unfavorable residues until proper folding or assembly buries them within the protein core (Figure 6f). Recent studies in eukaryotes have begun to elucidate how such intramembrane chaperones operate.

One example is the PAT complex, a membrane-embedded chaperone associated with the MPT and composed of CCDC47 and Asterix. Asterix directly binds TMs via its amphiphilic substrate-binding surface, with a preference for marginally hydrophobic TMs. This interaction is diminished upon translation completion. These findings support a model in which the PAT complex acts as an intramembrane chaperone, shielding TMs with exposed hydrophilic side chains.^33,251^

EMC is an additional intramembrane chaperone, acting separately from the MPT. In addition to its well-characterized insertase activity, the EMC has a broad repertoire of multispanning substrates, with preferential binding to TMs enriched in bulky or charged residues.^154,155,280,281^ Structural studies have revealed that the membrane-embedded region of EMC contains two cavities: a hydrophilic groove associated with its insertase function, and a lipid-filled hydrophobic cavity on the opposite face that may support additional roles.^141,144–146^ Indeed, cryo-EM analysis of human EMC in complex with a partially assembled calcium channel showed that channel subunits interact with EMC’s hydrophobic cavity, suggesting that EMC can act as a chaperone to facilitate channel assembly.^152^ Recent work further demonstrated that the hydrophobic cavity of EMC, and more specifically the EMC1 subunit, is important for binding of marginally hydrophobic TMs. Intriguingly, EMC1 was shown to interact with the hydrophobic, rather than the polar residues of such TMs, resulting in modulation of their membrane orientation that might affect their assembly with other TMs of the protein.^145,154^

The PAT complex and EMC lack obvious bacterial homologues, and the mechanisms governing shielding of marginally hydrophobic TMs in bacteria remain largely uncharacterized. Experimental evidence suggests that YidC acts not only as an insertase but also as a chaperone, assisting the folding or assembly of several substrates.^163–166,182^ However, a specific role in chaperoning marginally hydrophobic TMs has not been demonstrated. Interestingly, the hydrophobicity threshold for SecYEG-mediated TM insertion into the *E. coli* membrane is lower than that of the ER Sec61, suggesting that marginally hydrophobic TMs might pose less of a challenge in prokaryotes.^259^

Overall, the insertion of polar or marginally hydrophobic helices depends primarily on structural and sequence-encoded adaptations, as well as chaperones that transiently protect them until folding or assembly is complete.

### 4.4 How Much Insertion Occurs without Machinery?

While insertion is generally a machinery-dependent process, several proteins have been observed to integrate into membranes without assistance. The most established examples are proteins that have specifically evolved to insert spontaneously, including membrane-active toxins that must penetrate membranes from the extracellular side,^282^ viral fusion proteins,^283,284^ antimicrobial peptides,^285^ and certain synthetic or model peptides^216,286^ (see also the review in this issue by Francisco Barrera and colleagues). These examples demonstrate that spontaneous insertion is chemically possible, yet they raise a broader question: to what extent could such unassisted insertion contribute to the biogenesis of physiological membrane proteins?

Answering this question has proven difficult. Over the years, several proteins were believed to insert spontaneously until their corresponding insertion factors were identified. This is particularly true for simple single- or double-spanning proteins such as mitochondrial^287,288^ or ER-localized^289–292^ tail-anchored proteins, as well as small bacterial membrane proteins.^293–295^ Their apparent spontaneity was inferred from two main observations: (i) insertion persisted in membranes depleted of all insertion factors known at the time, and (ii) the proteins integrated efficiently into pure liposomes devoid of any proteins. While these findings were convincing at the time, later work revealed that these substrates rely on dedicated machineries that were simply unidentified then: in bacteria on YidC,^242^ in the ER on the GET and EMC pathways,^77,78^ and in the mitochondrial outer membrane on MIM1, Mtch2, or related proteins.^84,85,296^

These examples illustrate the challenge of demonstrating genuine spontaneity. *In vivo*, it is nearly impossible to rule out the contribution of unknown factors; simultaneously, *in vitro*, conditions that enable insertion into pure lipid bilayers may not reflect the constraints of cellular membranes. Consequently, many apparent cases of spontaneous insertion have later been reclassified as assisted. Yet this does not exclude the possibility that spontaneous insertion occurs in cells; it only illustrates how difficult it is to demonstrate conclusively.

Residual insertion observed in depletion or knockout experiments provides further hints for spontaneous insertion. When a key insertion factor is absent, the efficiency of insertion typically drops but is rarely abolished, often remaining in the 30–50% range.^91,149,214,217^ Whether this remaining activity reflects genuine spontaneous insertion or the action of redundant or as-yet-unidentified pathways remains unclear. Either way, these findings suggest that unassisted insertion may be more common than appreciated, and that even the most sophisticated insertion networks may coexist with a background level of spontaneous membrane integration.

## 5 When Things Go Wrong: Handling Misinsertion

Even with the elaborate network of pathways that evolved to accommodate diverse TM sequences, insertion occasionally fails. Misinsertion, an event in which one or several TMs of a protein fail to insert properly, can compromise protein function and cell health. Since misinserted MPs are rarely detected in healthy cells,^297^ membrane insertion might appear nearly error-free. Yet such events may actually occur frequently, given the constant synthesis of challenging-to-insert TMs. These mistakes are typically handled by quality control mechanisms that either reinsert the protein, restoring its proper topology and localization, or eliminate the defective species through degradation.

The frequency of misinsertion depends on the specific protein and TM. Hydrophilic TMs, for instance, are more prone to failures,^10,46,268,298,299^ and pathogenic mutations that increase TM hydrophilicity can perturb insertion and protein folding.^280,300^ Because misinserted species are rapidly degraded, they often escape detection. But they become apparent under specific conditions: when tracking immature proteins during biogenesis,^46,161,277,301^ when quality control pathways are disrupted,^268,302^ or when misfolded mutant proteins overload the quality control system and accumulate to measurable levels.^214,280,300,303^ Notably, quality control capacity declines with age, raising the possibility that misinserted proteins may accumulate in older cells, as seen in retinal degeneration caused by mutant rhodopsin.^304^

Misinsertion can take several forms. A protein may fail to insert entirely, remaining in the cytosol,^305,306^ or it may be mistargeted and inserted into the wrong membrane.^51,307,308^ While such cases could be classified as mistargeting, we consider them here as misinsertion events, since correcting them involves reinsertion into the correct membrane. Another failure mode is incorrect topology; for example, a single-pass protein can insert with the wrong orientation. In multispanning proteins, the outcome may become more complex, as failure to insert one TM properly can misorient adjacent helices due to the zigzag pattern of membrane traversal,^264,280,301,309,310^ disrupting the overall topology.

Regardless of the precise defect, misinsertion is typically detrimental. Mislocalized proteins cannot function at their intended site and may aggregate when exposed to the cytosol.^311^ Multispanning proteins with misinserted or misoriented TMs are unlikely to fold and function properly.^214,280,300^ These species can become cytotoxic, necessitating their rapid elimination. Yet degradation leaves only a brief window during which misinserted proteins might be rescued and reinserted. Both reinsertion and degradation therefore need to occur swiftly, requiring fast recognition and handling.

### 5.1 Detecting Misinsertion

Regardless of whether a misinserted protein is eventually degraded or rescued, the first essential step is its detection. Although the full range of detection mechanisms is not yet fully characterized, several distinct strategies have already emerged.

First, misinsertion can be detected indirectly by sensing protein misfolding. This is especially important for multispanning proteins, where a misinserted TM can cause misfolding within the lipid bilayer. The misfolding defects can be sensed by membrane-embedded quality control factors, often leading to the extraction of the protein from the membrane and degradation. The best studied quality control systems are found in the eukaryotic ER (namely, ER-associated degradation)^312^ (see review by Sonya Neal and colleagues in this issue), which also retains misfolded MPs. However, other systems that handle misfolded MPs are found in essentially all studied biological membranes.^313–316^ The details of misfolded MP recognition are still being uncovered and are beyond the scope of this review. However, several general principles have emerged. For instance, misfolded transmembrane regions often expose polar or charged residues to the lipid environment, a deviation from the normally hydrophobic TM surface.^152,251,317–320^ In addition, misfolded proteins may possess unstable helices that may unwind and be cleaved by intramembrane proteases.^321–327^ When a protein is a part of a multisubunit complex, misfolding or mistargeting may prevent complex assembly, leaving the orphan protein susceptible to quality control.^277,307,308^

However, misinsertion can also be sensed directly. One major detection route relies on recognizing TMs in the *cytosol*. For example, if a single-pass membrane protein fails to insert, its hydrophobic TM segment remains exposed to the aqueous environment. Such exposed hydrophobic regions are classical triggers for cytosolic quality control systems. Soluble chaperones can bind these hydrophobic regions, preventing aggregation and targeting the client for degradation.^305,306,328^ Similar principles apply to the ER lumen, where TMs translocated into the lumen instead of inserting into the membrane can be captured by the luminal chaperone BiP.^280,298,329^ Additionally, targeting factors such as SRP, GET3, NAC, and others function as TM-binding chaperones during normal biogenesis^59^ (see the above section 3.1 on targeting). Their ability to recognize TMs in the cytosol means they can possibly act as *sensors* of insertion failure, capturing uninserted proteins and delivering them back to the membrane for a second insertion attempt.

In multispanning membrane proteins, misinsertion creates a misfolded species with different challenges for quality control. Instead of being fully exposed in the cytosol, the misinserted TM remains tethered to the membrane by the rest of the protein. Depending on its sequence, it may sample the aqueous environment, the bilayer interface, or interact with membrane-bound chaperones.^18,25,329^ The membrane anchoring can partly stabilize the misinserted TM and reduce its exposure to the degradation machinery.^237^ Nonetheless, misinsertion defects are eventually recognized and targeted for removal. For instance, TMs exposed to the ER lumen can be captured by the chaperone BiP,^280,298,329^ directing the protein to ER-associated degradation. Additionally, Oxa1-family insertases contain substrate-binding domains capable of binding hydrophobic regions outside the membrane.^127,134,146^ Although their roles in handling misinsertion are not well characterized, they may help serve as an initial capture point for uninserted TMs.^329^

Misinserted TMs may also be sensed within the membrane. For example, single-pass tail-anchored proteins can frequently mislocalize between the mitochondrial and ER membranes. These TMs have somewhat divergent sequences, with mitochondrial TMs tending to be less hydrophobic and containing a C-terminal exocytosolic positively charged tail.^88^ Quality control mechanisms can utilize these properties to recognize and bind mistargeted TMs presenting these features in the incorrect membrane.^302,330,331^ Similar principles may be utilized to recognize misoriented TMs, since oppositely oriented TMs (N-exo vs N-cyt) have somewhat different properties. For example, misoriented TMs may disobey the ‘positive-inside’ rule, exposing positively charged residues to the luminal side of the ER, which recruits quality control factors that can repair the protein.^264,302,332^

### 5.2 Repairing Misinserted Proteins

Evidence that misinsertion is common comes from recent discoveries of mechanisms that correct it. If misinsertion were rare, cells could simply degrade the occasional faulty protein. Instead, the growing number of identified repair pathways suggests that misinsertion often occurs as a temporary intermediate on the way to proper biogenesis.

The clearest examples of repair come from recent studies of mistargeted single-pass proteins, both C-terminal tail-anchored and N-terminal signal-anchored. Such proteins often mislocalize between the ER, mitochondria, and, to some extent, peroxisomes. In the ER membrane, mitochondria-destined proteins are recognized by the conserved P-type ATPase Spf1 (ATP13A1 in humans),^302,332–334^ which uses ATP to extract the TM from the membrane, allowing another round of targeting to mitochondria. In the outer mitochondrial and peroxisomal membranes, a distinct mechanism operates: the AAA+ ATPase Msp1 (ATAD1 in humans) pulls mistargeted proteins out of the membrane and releases them into the cytosol for retargeting.^308,335,336^ Blocking either system causes a marked accumulation of proteins in the wrong organelle, indicating that many proteins initially mistarget. These factors are thus not rare “emergency” systems, but integral components of the cellular targeting network.^307,333,335,337,338^

The ability of the above retargeting pathways to handle multispanning proteins is less well understood, but a pathway capable of doing so has been identified. The ERSURF pathway salvages certain mitochondrial carrier proteins, multispanning proteins of the inner mitochondrial membrane, that can initially be targeted to the ER.^339,340^ ERSURF relies on Djp1, an ERanchored Jdomain chaperone that likely binds these clients and keeps them unfolded and translocation-competent.^339,340^ It remains unclear whether their transmembrane segments actually insert into the ER membrane and require dislocation, or whether the TMs stay chaperone-bound at the membrane surface until transfer.

Another error that may occur is the misorientation or misinsertion of TMs in multispanning proteins. These events are difficult to study at scale, but some proteins are known to be initially inserted with partially incorrect topology,^46,262,301,341^ and certain topological rearrangements can take place long after translation.^46,301,341–344^ Some of these changes may occur spontaneously, likely driven by unstable or “frustrated” topologies.^46,309,310,345^ Others, however, probably require active assistance to move highly hydrophilic loops or tails across the membrane. Although this area remains less explored, recent findings have revealed ER mechanisms that can correct such errors. Spf1 (ATP13A1 in humans) can identify misoriented TMs that violate the ‘positive-inside’ rule and eject them from the membrane into the cytosol.^264,332,333^ Though not characterized in detail, Spf1 lacks a transmembrane pore and can likely only translocate short loops flanking the TM. However, recent findings suggest that even large soluble domains can reorient across the membrane via unknown mechanisms.^346,347^

Regardless of the mechanism of TM ejection, insertases such as EMC and YidC can then likely bind TMs exposed on the cytosolic side and insert them into the membrane.^91,151,214^ Several insertases are also able to bind misfolded proteins,^91,152,165,166^ raising the possibility that misinserted proteins, which likely grossly misfold, can recruit insertases to help restore their correct topology. Spf1/ATP13A1 has recently been shown to form complexes with both EMC and Sec61, suggesting coordinated action of the dislocase with the insertion machinery.^332,348^ The emerging field of membrane protein chaperoning and quality control will likely uncover more such repair activities.

## 6 Conclusion and Future Perspective

Together, the findings discussed here show that membrane insertion is not a rigid or error-free process, but a dynamic and self-correcting system. The sequence cues and physical principles that guide proper TM insertion also enable the surveillance and repair of errors. Membrane protein insertion thus forms a continuum, from targeting and insertion to quality control and repair, ensuring that even an inherently error-prone process remains remarkably reliable in living cells.

However, despite rapid progress, important questions remain. At the level of targeting and pathway choice, much has been learned, yet we may still not have identified all of the proteins and complexes that participate in insertion and its surveillance across different organisms. Even for the factors already identified, the picture remains incomplete, particularly with respect to the precise roles of the auxiliary factors, how they refine substrate selectivity, how alternative assemblies are dynamically engaged during biogenesis, and how broad principles defined for model substrates apply across the membrane proteome. It also remains unclear to what extent the principles inferred from individual studies apply beyond the specific experimental conditions, substrates, and reconstituted sets of auxiliary subunits in which they were defined. In addition, the extent to which these “rules of engagement” are conserved across organisms remains unknown. Other unresolved issues include how different insertion and auxiliary factors cooperate, compete, or exchange during the insertion of multispanning proteins or difficult substrates such as TMs with low hydrophobicity. More broadly, how do membrane properties, including lipid composition, packing, thickness, and asymmetry, reshape insertion efficiency and route choice across distinct cellular membranes?

Another major frontier concerns insertion failure and disease. What fraction of pathogenic membrane-protein variants acts primarily by perturbing insertion rather than later steps such as folding, assembly, trafficking, or stability? Can such variants be predicted from sequence, topology, and membrane context, particularly when mutations subtly alter TM hydrophobicity, charge distribution, or loop translocation demands? And when insertion is impaired, what is the most effective corrective strategy: stabilizing the defective TM during biogenesis, rerouting the client to a more permissive pathway, modulating the membrane environment or auxiliary machinery, or promoting repair and reinsertion? Addressing these questions will be essential for moving from a mechanistic description of insertion pathways to a predictive understanding of membrane-protein biogenesis in health and disease.
